# Effects of Endophytic Fungus *Setophoma terrestris* on Growth of *Panax notoginseng* and Its Rhizosphere Soil Microorganisms

**DOI:** 10.3390/life15091353

**Published:** 2025-08-27

**Authors:** Huali Li, Jian Liu, Yajiao Sun, Mengyao Wang, Shuwen Liu, Yunqiang Ma, Junjia Lu

**Affiliations:** 1 College of Landscape Architecture and Horticulture Sciences, Southwest Forestry University Sciences, Kunming 650224, China; 15912938064@163.com (H.L.); jian927520@163.com (J.L.); 18087323192@126.com (Y.S.); 17587020327@163.com (M.W.); 15887642939@163.com (S.L.); 2 Yunnan Key Laboratory of Forest Disaster Warning and Control, Southwest Forestry University, Kunming 650224, China; 3 Yunnan Provincial Key Laboratory for Conservation and Utilization of In-forest Resource, Southwest Forestry University, Kunming 650224, China; mayunqiang@swfu.edu.cn

**Keywords:** *Setophoma terrestris*, *Panax notoginseng*, growth-promoting function, rhizosphere soil microorganisms, metabolomics

## Abstract

To investigate the effects of the endophytic fungus *Setophoma terrestris* (isolated from *Panax notoginseng* roots) on the growth and rhizosphere microbiota of understory-cultivated *P. notoginseng*, we prepared liquid and solid fermentates of the fungus and applied them separately via irrigation. Rhizosphere soil of *P. notoginseng* was subjected to non-targeted metabolomics and microbiome sequencing for detection and analysis. Relative to the control, *P. notoginseng* treated with liquid and solid fermentates exhibited increases in plant height (3.5% and 0.7%), chlorophyll content (23.4% and 20.4%), and total saponin content (14.6% and 17.0%), respectively. Non-targeted metabolomics identified 3855 metabolites across 23 classes, with amino acids and their derivatives (21.54%) and benzene derivatives (14.21%) as the primary components. The significantly altered metabolic pathways shared by the two treatment groups included ABC transporters, purine metabolism, and the biosynthesis of various other secondary metabolites. Exogenous addition of *S. terrestris* significantly affected the composition of the rhizosphere soil microbial community of *P. notoginseng* and increased the relative abundance of genera such as Bradyrhizobium. In conclusion, the endophytic fungus *S. terrestris* enhances *P. notoginseng* growth and modulates both rhizosphere soil metabolites and microbial abundance. This study can provide certain data support for research on endophytic fungi of *P. notoginseng*.

## 1. Introduction

*Panax notoginseng* (Burk.) F. H. Chen, a perennial root herb in the genus *Panax* (Araliaceae), is a valuable traditional Chinese medicinal plant with therapeutic properties primarily derived from secondary metabolites, including saponins and flavonoids [1,2]. Current *P. notoginseng* cultivation is plagued by continuous cropping barriers induced by autotoxicity and pathogen accumulation [3], which impair both yield and quality of medicinal materials and restrict large-scale cultivation; notably, endophytic bacterial biofertilizers have been shown to mitigate such obstacles. Endophytic fungi further alleviate these barriers by modifying soil physicochemical properties, enhancing microbial diversity, and suppressing pathogen proliferation via secondary metabolite secretion and nutrient competition [4,5]. Plant endophytic fungi, symbiotic microorganisms colonizing host tissues, are pivotal for host adaptation to environmental stresses, as they modulate plant metabolic networks through secretion of secondary metabolites (e.g., terpenoids, alkaloids) [6,7,8]. Recent studies on *P. notoginseng* endophytes have yielded notable findings: Liu et al. (2016) isolated 11 endophytes strains from seeds and roots, including one saponin-producing isolate [9]. Huang et al. (2022) identified 17 endophytic bacteria and 45 fungi from across 42 genera from *P. notoginseng* tissues [10]; and Zhang et al. recovered 22 endophytic actinomycetes (encompassing *Streptomyces*, *Flavihumibacter*, *Actinomycetospora* and *Nocardiopsis*) from whole plants across two regions using three isolation techniques [11].

Several studies have shown that endophytes exhibit a positive promoting effect on plant growth and development [12]. Huang reported that *P. notoginseng* endophytes synthesize growth regulators (e.g., indole-3-acetic acid (IAA), gibberellins) to promote host growth [10], while Linfeng et al. [13] isolated the endophytic fungus *Phoma medicaginis* from *Lycium chinense*, whose fermentation broth increased leaf number, root length, and stem length in tissue-cultured seedlings to varying degrees. *Aspergillus oryzae* AVNF4, isolated from the rhizomes of *Curcuma longa* by Sanneboyina and Audipudi, not only inhibits *Fusarium oxysporum* (the causal agent of tomato wilt) but also promotes the growth of tomato seedlings by secreting IAA, solubilizing inorganic phosphorus, and producing volatile organic compounds (e.g., 1,3-dioxolane, oleic acid). After treatment with its liquid microbial agent, the germination rate, plant height, and root biomass of tomatoes were significantly increased [14].

The rhizosphere microbial community is a critical driver of plant health, as it inhibits pathogen invasion and proliferation, sustains host fitness, and stabilize the microecosystem [15,16]. Endophyte inoculation directly or indirectly modulates soil microbial community structure, diversity, and functional composition, thereby affecting plant growth [17,18]; for example, the cotton stems endophyte *F. equiseti* reshapes soil microbial communities, enriches beneficial bacteria taxa, and reduce verticillium wilt incidence in cotton [19]. Endophytic fungi can also affect soil chemical properties. Research has revealed that endophyte inoculation significantly changes soil pH, electrical conductivity (EC), and nutrient availability. Under drought stress, endophytic fungi ameliorate the soil chemical environment via reduced EC, elevated nitrate and ammonium nitrogen contents, and enhanced the release of phosphorus and potassium, thereby boosting sunflower drought tolerance. Endophytic fungi may also regulate soil nutrient cycling via secretion of organic acids and enzymes [20].

Microbial fertilizers produced using plant growth-promoting endophytes can improve the soil environment and promote crop growth through metabolic activities. Within microbial communities, phosphorus-solubilizing, potassium-solubilizing, and nitrogen-fixing bacteria decompose soil-insoluble phosphorus and potassium, fix atmospheric nitrogen, transform recalcitrant nutrients into plant-available forms, promote plant growth, and reduce dependence on chemical fertilizers.

Using healthy *P. notoginseng* plants as test materials, we isolated and identified culturable endophytic fungi via tissue isolation, then assessed their growth-promoting activity and inhibitory effects against 5 pathogenic fungi, ultimately screening a strain with superior growth-promoting and disease-resistant traits. Two biofertilizer formulations of this strain were prepared, administered to understory vegetative-stage *P. notoginseng*, and followed by harvesting of plants and rhizosphere soil to assess the strain’s impacts on host growth, rhizosphere metabolites, and soil microorganisms.

## 2. Materials and Methods

### 2.1. Test Materials

Test strain: Endophytic fungi were isolated from the roots of *P. notoginseng* using the tissue isolation method, and screening for growth-promoting functions (such as siderophore production, and IAA production) was conducted. A strain with excellent growth-promoting capabilities was selected for this experiment. It was identified as *S. terrestris* through molecular biological identification combined with morphological characteristics, and its accession number in the NCBI database is PV992114.

Test plants: Three-year-old understory *P. notoginseng* plants from an understory planting base of *P. notoginseng* in Xundian County, Yunnan Province (22°37′20.93″ N).

### 2.2. Experimental Design

A completely randomized design included three groups: solid fermentate inoculation (SF, 10 g/plant, uniformly applied to 3–4 cm root-zone soil, 10 replicates), fermentation broth (LF, 20 mL/plant, syringe-irrigated at roots, 10 replicates), and clear water treatment (CK, 20 mL/plant via syringe, 10 replicates).

The control group received 20 mL of water via syringe per plant (10 replicates), with all treatments initiated on 10 April 2023, and repeated every 15 days for a total of 6 applications, concluding on 9 June 2023. During non-treatment periods, all *P. notoginseng* plant sreceived uniform management with adequate irrigation and were harvested along with rhizosphere soil 15 days after the final treatment for the determination of various indicators.

### 2.3. Strain Fermentation Treatment

#### 2.3.1. Preparation of Fermentates

Liquid fermentation: A 6-mm mycelial disc of *S. terrestris* was inoculated into Sabouraud Dextrose Agar with Yeast Extract liquid medium, shaken at 121 r/min for 10 days at room temperature, centrifuged to harvest the supernatant, and adjusted to a concentration of 1 × 10^8^ CFU·mL^−1^ for the fungal suspension. SF: Components for SFs were thoroughly mixed, aliquoted, sterilized, inoculated with cultured seed liquid, and incubated in a constant-temperature incubator until the mycelia fully colonized the tissue culture flask [21].

#### 2.3.2. Sample Collection and Pretreatment

Plants: First, aboveground height of *P. notoginseng* plants was measured, followed by root system collection with preservation of plant integrity for subsequent analyses.

Soil: Rhizosphere soil adhering to *P. notoginseng* roots was collected using the shaking-off method, sieved through a 2-mm mesh, and aliquoted into fractions: one stored at −20 °C for immediate root metabolite analysis, another at −20 °C for soil chemical property assessment, and the final fraction at low temperature for subsequent supplementary assays.

### 2.4. Determination of Plant Biomass

#### 2.4.1. Fresh and Dry Weights

Collected *P. notoginseng* plants were cleaned to remove dust, air-dried briefly, and then weighed for fresh weight; subsequent drying at 80 °C was followed by dry weight determination.

#### 2.4.2. Determination of Chlorophyll Content

A 0.1-g sample of *P. notoginseng* leaves was placed in a mortar, and a small amount of quartz sand, calcium carbonate powder, and 1 mL of 80% acetone was added. The mixture was ground to a homogenate in the dark. The homogenate was transferred to a 25-mL brown volumetric flask, with residual homogenate on the mortar and pestle rinsed into the flask using 80% acetone; the mixture was then brought to 25 mL with 80% acetone, vortexed thoroughly, and centrifugation at 8000 rpm for 5 min. The extract was poured into a cuvette, with 80% acetone as the blank for zeroing, and absorbance was measured at 663 nm and 645 nm [22].

#### 2.4.3. Determination of Total Saponins

The main roots of *P. notoginseng* were dried, ground, and passed through a 100-mesh sieve. A 1-g sample was weighed into an Erlenmeyer flask, supplemented with 50 mL of 80% methanol, vortex-mixed, and subjected to ultrasonic extractor at 60 °C (465 W) for 40 min. A 40-µL aliquot of the sample solution was transferred to a 96-well microplate, evaporated to dryness in an 80 °C constant-temperature water bath, then supplemented with 20 µL of 5% vanillin-glacial acetic acid solution (5 g vanillin dissolved in 100 mL glacial acetic acid) and 80 µL of perchloric acid. The mixture was heated in a 60 °C water bath for 15 min, cooled in an ice bath for 5 min, then supplemented with 40-µL glacial acetic acid to terminate the reaction, and its absorbance was measured at 540 nm [23]. Meanwhile, the absorbance values of *P. notoginseng* total saponin solutions (0, 50, 100, 150, 200, 250 µg/mL) were determined under identical conditions, and a standard curve (X: concentration; Y: absorbance) was generated to quantify PNLS (*P. notoginseng* total saponins) content in the samples.

### 2.5. Determination of Metabolites in Rhizosphere Soil

*Panax notoginseng* rhizosphere soil samples were stored on dry ice and shipped to Wuhan MetWare Biotechnology Co., Ltd. (Wuhan, China) for LC-MS metabolomics analysis (3 replicates per treatment). Briefly, 250 mg (±1 mg) aliquots were weighed into a 2 mL centrifuge tube, supplemented with 500 μL of pre-cooled (−20 °C) 70% methanol–water internal standard extraction solution, and vortexed for 3 min (steel balls were added for re-vortexing 3 min if samples remained undispersed). After ultrasonic treatment in an ice-water bath for 10 min, the mixture was vortexed for 1 min and then allowed to stand at −20 °C for 30 min. Centrifugation was performed at 12,000 r/min at 4 °C for 10 min, and the supernatant was passed through a 0.22-μm PTFE filter membrane into the liner of a brown sample vial. Equal volumes of supernatant from all samples were mixed to form a quality control (QC) sample before instrumental analysis [24,25].

Chromatographic separation was performed using an ultra-high-performance liquid chromatograph (LC-30A) fitted with a Waters ACQUITY Premier HSS T3 Column. The mobile phase consisted of 0.1% formic acid/water (A) and formic acid/acetonitrile (B), with elution via a defined gradient. The column initial temperature was 40 °C, the flow rate was 4 mL/min, and the injection volume was 4 μL. Mass spectrometry employed a TripleTOF 6600+ for primary and secondary mass spectral data collection, with dynamic exclusion of redundant MS/MS information.

Data processing: After converting raw data to mzXML format, peak detection, alignment, and retention time correction were performed using the XCMS program. Peak areas were corrected using the “SVR” algorithm, and peaks with a detection rate lower than 50% were excluded. Metabolite annotations were retrieved via searches of relevant databases. Principal component analysis (PCA) was performed using Principal Component Analysis (PCA) was performed using the prcomp function in R (version 4.1.2) (with data normalization). Hierarchical cluster analysis (HCA) was conducted using the cor function in R (version 4.1.2) based on the Euclidean distance matrix (Ward.D2 method). Differential metabolites were screened based on VIP > 1 (from PLS-DA) and *p* < 0.05 (from Student’s *t*-test).

### 2.6. Determination of Soil Physicochemical Indicators

#### 2.6.1. Determination of Soil Total Nitrogen (TN) and Total Phosphorus (TP) Contents

Rhizosphere soil of *P. notoginseng* was air-dried, ground, and passed through a 100-mesh sieve. A 0.5-g sample was weighed into a digestion tube, and 5 mL of H_2_SO_4_ was added. The digestion tube was placed in a graphite furnace, heated slowly to 280 °C, held for 5 min, removed to cool, then returned, heated to 370 °C, held for 5 min, and cooled again. The cap was opened, and 10 drops of H_2_O_2_ added, and the tube digested at 370 °C for 12 min before removal and cooling. Digestion continued at 370 °C with sequential H_2_O_2_ additions (8, 6, 4, 2 drops) until the solution was colorless and transparent. Post-digestion, the mixture was cooled. The digestion tube was rinsed 3 times with deionized water, and the contents were filtered and transferred to a 100-mL volumetric flask for volume adjustment [26]. A sample-free blank control was included. The contents of total nitrogen and total phosphorus were determined using a flow analyzer (SEAL Analypay AA3) and calculated accordingly.

#### 2.6.2. Determination of Total Potassium (TK) Content in Soil

*Panax notoginseng* rhizosphere soil was air-dried, ground, and sieved through a 100-mesh sieve. A 0.5 g soil sample was weighed into a digestion tube, with a small volume of deionized water rinsing wall-adhered soil particles to the tube bottom. In a fume hood, 6 mL of HCl and 4 mL of HNO_3_ were added to the tube. After capping tightly, it was placed in a graphite furnace and heated at 105 °C for 60 min. Once cooled to near room temperature, 2 mL of HClO_4_ and 1 mL of hydrofluoric acid were added. The mixture was heated at 170 °C for 150 min until the acid emitted white fumes and the sample became colorless/viscous, indicating digestion completion. The digested sample was cooled to room temperature, and the digestion tube was rinsed 3× with deionized water, and the rinsate transferred to a 50-mL volumetric flask and brought to volume with deionized water. A blank control (without soil sample) was set simultaneously. The total potassium content was determined using the AA-6300C flame photometer by atomic emission spectrometry [27].

#### 2.6.3. Determination of Soil Nitrate Nitrogen (NO_3_^−^-N) and Ammonium Nitrogen (NH_4_^+^-N) Contents

*Panax notoginseng* rhizosphere soil was air-dried, ground, and sieved through a 20-mesh sieve. A 10-g aliquot was weighed into a 300-mL Erlenmeyer flask and supplemented with 100 mL of 1 mol/L potassium chloride solution. The mixture was shaken on a shaker for 60 min and then allowed to stand for 30 min. The sample solution was filter-paper filtered, and the filtrate was stored in a 50-mL centrifuge tube. A blank control was set simultaneously. Determination was performed using an automatic continuous flow analyzer (Seal AutoAnalyzer AA3) [27], following the procedure: preparation of standard samples and cleaning solution → instrument startup → program selection → determination of standard sample concentration → determination of sample concentration. Finally, the soil organic matter content was calculated using the formula.

#### 2.6.4. Determination of Soil Organic Matter (SOM) Content

Rhizosphere soil of *P. notoginseng* was air-dried, ground, and sieved through a 100-mesh screen. A 1-g aliquot was transferred to a 10-mL hard test tube, supplemented with 0.8 mol/L potassium dichromate solution to half-volume, and then brought to the 10-mL mark with concentrated sulfuric acid, capped tightly, and vortexed thoroughly. The test tubes were batch-placed on a test tube rack, heated to boiling (180–195 °C) for 5 min, and then removed and cooled. The entire sample solution was transferred to a 250-mL Erlenmeyer flask, with residual test tube liquid rinsed into the flask using deionized water. Upon reaching ~70 mL, 2–4 drops of o-phenanthroline indicator were added. The solution was titrated with a standard ferrous sulfate solution until it turned brick-red [28]. The volume used (V) was recorded, and the organic carbon content was calculated using the formula.

#### 2.6.5. Determination of Soil pH Value

Soil pH was determined with a pH meter with a soil-to-water mass ratio of 2.5:1 [29]. Each treatment included three replicates. For each replicate, 5 g of fresh *P. notoginseng* rhizosphere soil was weighed, 25 mL distilled water added, and the mixture shaken for ~5 min on a shaker before standing to allow phases separated. After filtration with filter paper, the pH meter was calibrated with acidic and alkaline standards prior to measurement. Finally, the soil pH value was determined and recorded.

### 2.7. Determination of Rhizosphere Soil Microbial Diversity

Rhizosphere soil samples of *P. notoginseng* were stored in dry ice and then shipped to Wuhan MetWare Biotechnology Co., Ltd. for analysis (3 replicates per treatment). DNA was extracted using the OMEGA kit (per manufacturer’s protocol), followed by PCR amplification, product purification, library preparation and quality inspection, and paired-end high-throughput sequencing on the Illumina PE150 platform (NovaSeq) to generate raw data [30].

The sequencing data underwent quality control and assembly to generate Clean Tags, with valid data obtained post-chimera filtering. Denoising valid data yielded Amplicon Sequence Variants (ASVs), which informed species annotation and abundance distribution analysis. Meanwhile, ASV abundance and Alpha diversity were calculated, and Venn diagram analysis clarified the species richness and evenness within samples, as well as the shared and unique ASVs among samples [31]. Shannon index, Simpson index, Chao1 index, and ACE index are important indicators for describing the α-diversity of a community, each with distinct focuses. The Shannon index takes both species richness and evenness into account, with higher values indicating greater community diversity. The Simpson index reflects the dominance of the community; values closer to 1 suggest a more even distribution of species and less prominent dominant species. Both Chao1 and ACE indices are used to estimate the actual species richness of the community. Chao1 is calculated based on the number of rare species that appear 1–2 times, while ACE is more sensitive to rare species that appear ≤10 times. Both indices can correct for species omission caused by insufficient sample size, and their values are generally greater than the number of actually observed species. A larger difference between these values and the observed number indicates a higher possibility of undetected rare species.)

A phylogenetic tree was constructed by multiple sequence alignment of ASVs. Combined with dimensionality reduction analyses (e.g., Principal Coordinates Analysis [PCoA], sample clustering trees), community structure differences among samples or groups were explored. Statistical methods such as T-test assessed the significance of in species composition and community structure among grouped samples.

### 2.8. Data Analysis

The data were analyzed using statistical software Excel 2016 and SPSS 22.0. One-way ANOVA was applied to assess the statistical significance of differences among samples (*p* < 0.05), followed by Duncan’s multi-range test for multiple comparisons. Data visualization was performed using Adobe Photoshop 2023, Adobe Illustrator 2020, and GraphPad Prism 9.5.

## 3. Results and Analysis

### 3.1. Biomass

#### 3.1.1. Plant Height

**Figure 1:**
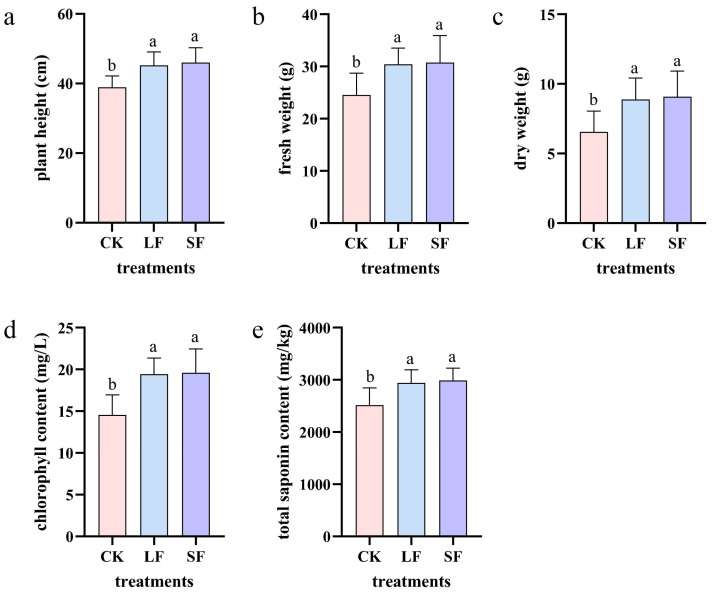
The impact of different treatments on the biomass of *Panax notoginseng*. (**a**) Plant height, (**b**) dry weight, (**c**) fresh weight, (**d**) chlorophyll content, and (**e**) total saponins. SF refers to the solid fermentation product treatment group, LF refers to the fermentation broth treatment group, and CK refers to the clear water treatment control group. The same applies to the figures below.

*Panax notoginseng* plant heights in both treatment groups exceeded that in CK: 38.94 cm), with LF and SF groups reaching 45.23 cm and 46.01 cm, respectively (Figure 1a); the rank order was SF > LF > CK.

#### 3.1.2. Fresh and Dry Weights

Fresh and dry weights of *P. notoginseng* in SF and LF groups exceeded those in CK (Figure 1b,c), with both parameters showing the same rank order: SF > LF > CK.

#### 3.1.3. Chlorophyll Content

Chlorophyll contents in SF and LF groups were significantly higher than in CK (Figure 1d), with the highest level in SF.

#### 3.1.4. Total Saponin Content

Total saponin contents in SF and LF groups were significantly higher than in the control (Figure 1e), with no significant difference between SF and LF.

### 3.2. Annotation and Evaluation of Metabolites in Panax notoginseng Rhizosphere Soil

#### 3.2.1. Qualitative and Quantitative Analysis of Metabolites

**Figure 2:**
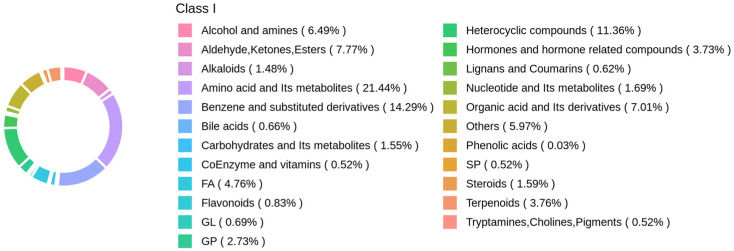
Circular Diagram of Primary Classification Composition of Rhizosphere Metabolites in *Panax notoginseng*.

To characterize metabolite variation across SF, LF, and CK groups, non-targeted metabolomics analysis identified 3855 metabolites spanning 23 categories (Figure 2). Dominant categories and their proportions included: amino acids and their metabolites (21.44%), benzene and its derivatives (14.29%), heterocyclic compounds (11.36%), aldehydes, ketones, and esters (7.77%), organic acids and their derivatives (7.01%), and alcohols and amines (6.49%). 5.97% others, 4.76% fatty acyls, 3.76% terpenoids, 3.73% hormones and hormone-related substances, 1.69% nucleotides and their metabolites, 1.59% steroids, 1.55% carbohydrates and their metabolites, 1.48% alkaloids, 0.83% flavonoids, 0.73% glycerophospholipids, 0.69% glycerolipids, 0.66% bile acids, 0.62% lignans and coumarins, 0.52% coenzymes and vitamins, 0.52% sphingolipids, 0.52% tryptamines, cholines and pigments, 0.03% phenolic acids.

#### 3.2.2. Principal Component Analysis (PCA) and OPLS-DA of Metabolites

**Figure 3:**
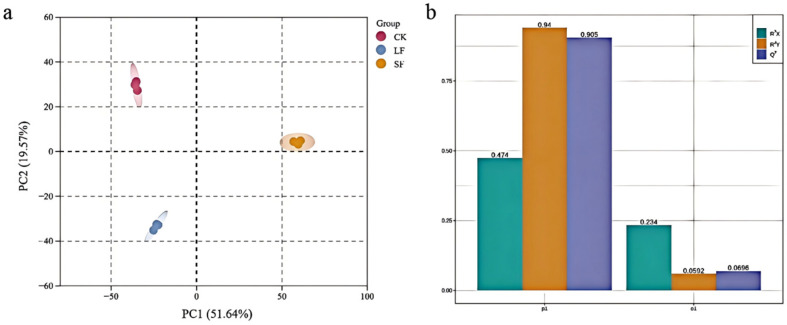
Principal Component Analysis and OPLS-DA Validation Plot of Total Soil Samples. (**a**) Principal Component Analysis, (**b**) OPLS-DA Validation Plot.

Principal component analysis was performed on rhizosphere soil metabolites across soil samples (Figure 3), showed nearly all samples within the 95% confidence interval. The first principal component accounted for approximately 51.64% of the metabolite variation, while the second principal component explained approximately 19.57%. LF and SF samples were distinctly separated from CK samples and clustered independently, suggesting endophytic fungi significantly altered rhizosphere soil metabolites.

Orthogonal partial least squares-discriminant analysis (OPLS-DA) with Euclidean distance fitting yielded Q^2^ > 0.50 and *p* < 0.05, confirming good model fitness and robustness for subsequent metabolite analysis.

#### 3.2.3. Cluster Heatmap Analysis of Metabolites

HCA was used to characterize differences in metabolite accumulation patterns across LF, SF, and CK samples. Based on this, metabolites were divided into four clusters:

Cluster I: Metabolites accumulated to the highest levels in CK, with only a small distribution in LF and SF.

Cluster II: Metabolites were most abundant in LF, moderately abundant in CK, and least abundant in SF.

**Figure 4:**
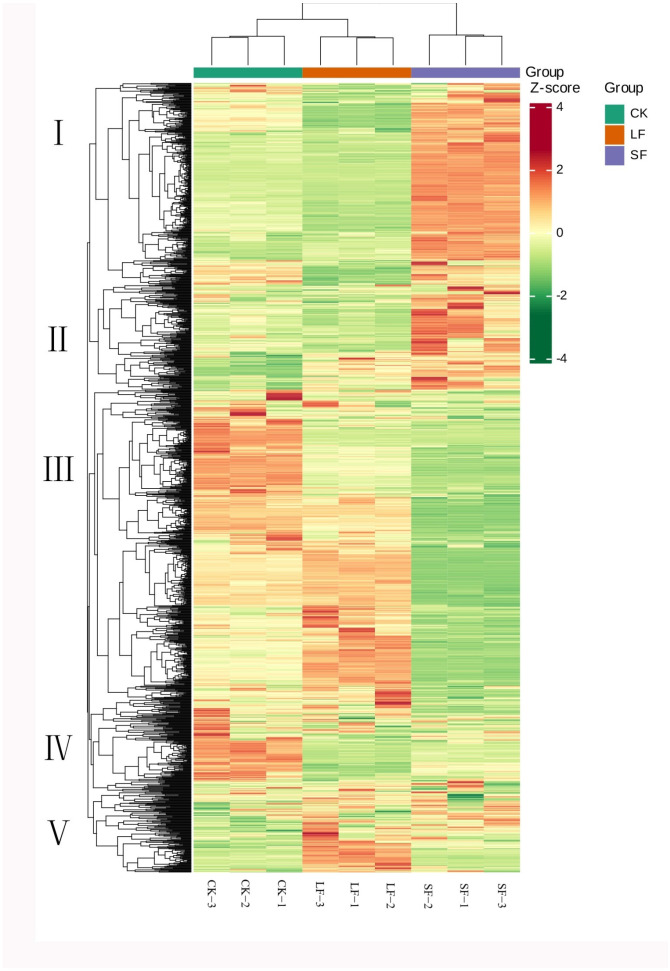
Clustering Heatmap of Metabolites.

Cluster III and IV: Metabolites displayed the highest content in SF, moderate levels in LF, and the lowest levels in CK (Figure 4).

#### 3.2.4. Volcano Plot Analysis of Differential Metabolites

**Figure 5:**
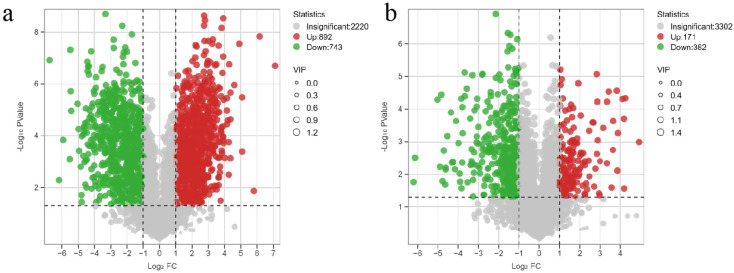
Volcano Plot of Up-regulated and Down-regulated Expressions of Differential Metabolites. (**a**) SF vs. CK, (**b**) LF vs. CK. Circles represent metabolites, with red and green indicating the increase or decrease of metabolites in the corresponding groups.

Differential metabolites were filtered based on fold change (FC) ≥ 2, VIP > 1, and *p* < 0.05 (Figure 5). Results revealed 1635 differential metabolites in SF vs. CK (892 upregulated, 743 downregulated) and 553 in LF vs. CK (171 upregulated, 382 downregulated).

**Figure 6:**
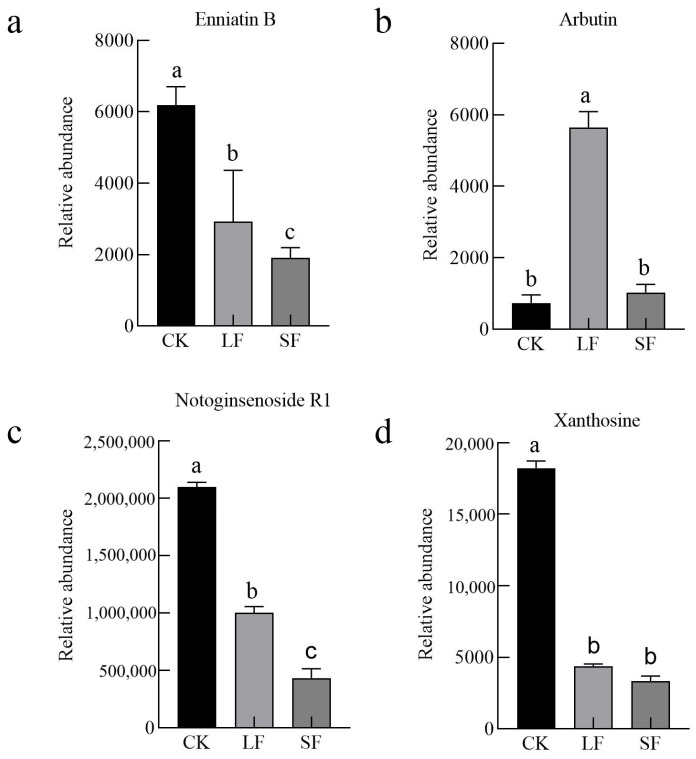
Analysis of Differential Metabolites in Partial Root Systems. (**a**) Enniatin B, (**b**) Arbutin, (**c**) Notoginsenoside R1, (**d**) Xanthosine.

Four metabolites overlapping and highly differential were screened across both comparisons (Figure 6), namely enniatin B1, arbutin, notoginsenoside R1, and xanthosine. Among them: Enniatin B1 is a toxic metabolite produced by *Fusarium* spp. Its content was reduced by both treatments, which may be attributed to the inhibition of *Fusarium* spp. by *S. terrestris*. Arbutin exhibits various pharmacological activities. LF treatment significantly increased its content, but its physiological effects on *P. notoginseng* require further research. Notoginsenoside R1, a key allelochemical in *P. notoginseng*, was downregulated by both treatments, potentially mitigating the species’ allelopathic autotoxicity. Xanthosine, which correlates negatively with *P. notoginseng* growth, was also reduced by both treatments.

#### 3.2.5. KEGG Functional Annotation

**Figure 7:**
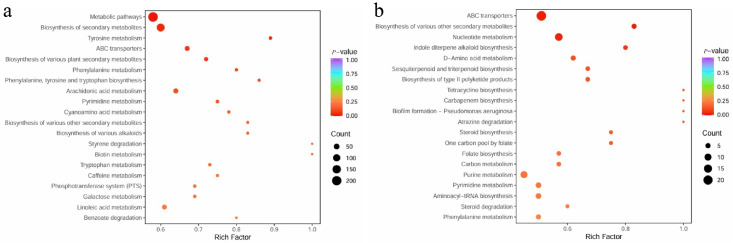
Enriched bubble plot of different metabolite pathway enrichment in different treatments. (**a**) Bubble plot of differential metabolite pathway enrichment under LF treatment; (**b**) Bubble plot of differential metabolite pathway enrichment under SF treatment.

Differential metabolites were mapped to the KEGG database followed by enrichment analysis (Figure 7). The results showed that: in the comparison between LF and CK, differential metabolites were primarily enriched in ABC transporters, nucleotide metabolism and synthesis, purine metabolism, miscellaneous secondary metabolite synthesis, and indole diterpenoid alkaloid biosynthesis; in the comparison between SF and CK, differential metabolites were predominantly enriched in metabolic pathways, synthesis of secondary metabolites, ABC transporters, purine metabolism, and synthesis of various other secondary metabolites. Notably, the overlapping metabolic pathways with significant differences between SF and LF treatments encompassed ABC transporters, purine metabolism, and the synthesis of miscellaneous secondary metabolites.

#### 3.2.6. Analysis of Key KEGG Metabolic Pathways

**Figure 8:**
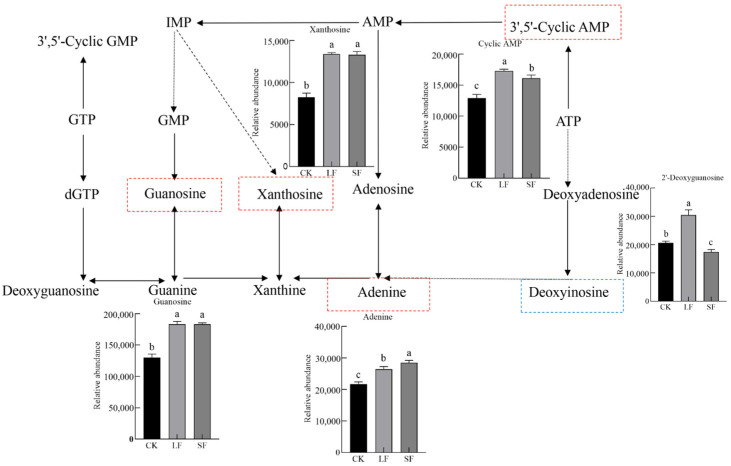
Purine metabolism pathway diagram.

A purine metabolic pathway, linked to both treatments, was selected for mapping (Figure 8). Results demonstrated that both LF and SF treatments increased the expression abundances of adenine, adenosine-3′-5′-cyclic monophosphate hydrate, xanthosine, guanosine, and other products in purine metabolism (acting as plant energy carriers and nitrogen sources), with LF treatment exhibiting higher abundances than SF treatment. Additionally, the abundance of 2′-deoxyinosine in the LF treatment was significantly greater than in the CK and SF treatments.

### 3.3. Annotation and Evaluation of Rhizosphere Soil Microorganisms of Panax notoginseng

#### 3.3.1. Effects of Growth-Promoting Fungi on Soil Nutrients and pH

Soil total nutrients reflect potential fertility, with total nitrogen content being intimately linked to plant growth. Relative to the control, the soil total nitrogen content in *P. notoginseng* increased following *S. terrestris* inoculation (across treatments), potentially due to the fungus-mediated enrichment of nitrogen-fixing bacteria. Total phosphorus and total potassium contents showed no significant changes: total phosphorus remained stable owing to the slow migration and transformation rates of phosphorus and the lack of exogenous supplementation, whereas total potassium maintained dynamic balance due to the absence of exogenous addition.

Soil nitrogen is crucial for plant growth. The SF treatment significantly increased ammonium nitrogen (NH_4_^+^-N) content (*p* < 0.05), whereas the LF treatment exhibited an increasing trend without statistical significance. Nitrate nitrogen (NO_3_^−^-N) content increased in all treatments, but no significant differences were detected.

The total phosphorus content in the rhizosphere soil of *P. notoginseng* did not show significant changes, which may be attributed to the slow migration rate of phosphorus in the soil environment and its relatively sluggish chemical transformation process. Additionally, no exogenous phosphorus supplementation measures were adopted throughout the experimental period; thus, different treatments with endophytes did not exert a significant impact on the total phosphorus content in the soil. Similarly, under the same experimental conditions, the total potassium content in the rhizosphere soil of *P. notoginseng* was not significantly affected by the exogenous addition of *S. terrestris*. This is because no exogenous potassium was added during the experiment, resulting in the soil potassium content maintaining a relatively stable dynamic equilibrium.

Soil organic matter serves as a key indicator of soil fertility. The LF treatment significantly increased organic matter content (*p* < 0.05), whereas the SF treatment showed an increasing trend without statistical significance. This could be attributed to the fungus’s capacity to modify microbial communities, thereby enhancing carbon input (Table 1).

Soil pH affects microbial activity and plant growth. Both LF and SF treatments significantly increased soil pH, indicating that this fungus might mitigate soil acidification.

In summary, exogenous addition of *S. terrestris* can promote soil nutrient cycling, increase nutrient contents, and improve soil fertility.

#### 3.3.2. Effects of Growth-Promoting Fungi on Taxonomic Composition of Rhizosphere Soil Microorganisms at the Phylum Level in Understory *Panax notoginseng*

**Figure 9:**
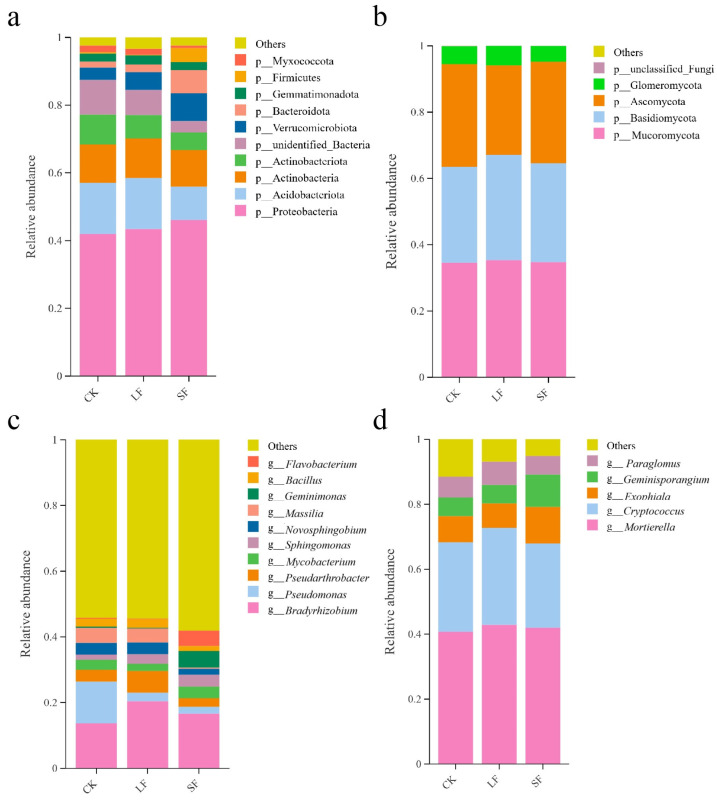
Community Composition of Soil Microorganisms at Phylum and Genus Levels under Different Treatments. (**a**) Community Composition of Bacteria at Phylum Level, (**b**) Community Composition of Fungi at Phylum Level, (**c**) Community Composition of Bacteria at Genus Level, (**d**) Community Composition of Fungi at Genus Level.

As shown in Figure 9, among the top 10 bacterial phyla by relative abundance in rhizosphere soil, the dominant phyla were Proteobacteria (42–46.1%), Acidobacteria (9.8–15.1%), and Actinobacteria (10.8–11.7%). Collectively, these three phyla contributed over 66% of the total bacterial community, thus dominating the assemblage. Other bacterial phyla included Myxomycota (0.6–1.9%) and Gemmatimonadetes (2.4–2.7%).

Compared with the control, all *S. terrestris* treatments increased the relative abundance of Proteobacteria, with a more pronounced elevation in the SF treatment than in the LF treatment. Additionally, the SF treatment decreased the relative abundances of Acidobacteria and Actinobacteria, while increasing those of Firmicutes and Verrucomicrobia.

Among the top 5 fungal phyla by relative abundance in rhizosphere soil, the dominant phyla were Basidiomycota (29–31.7%), Zygomycota (34.6–35.4%) (Note: The traditionally classified ‘Zygomycota’ has been revised due to its non-monophyletic nature. The taxa involved in this study are classified into Mucoromycota based on phylogenetic research (Spatafora et al.) [32], and will be referred to as Mucoromycota hereafter), and Ascomycota (27–30.9%). Together, these three phyla constituted over 90% of the total fungal community, thus dominating the assemblage. Another fungal phylum of note was Glomeromycota (0.1–0.3%).

Compared with the control, all *S. terrestris* treatments elevated the relative abundances of Mucoromycota and Basidiomycota, while the LF treatment decreased the relative abundance of Ascomycota.

#### 3.3.3. Effects of Growth-Promoting Fungi on Taxonomic Composition of Rhizosphere Soil Microorganisms at the Genus Level in Understory *Panax notoginseng*

As shown in Figure 9, genus-level bacteria comprised 11 major groups. Identifiable genera and their relative abundances were as follows: *Bradyrhizobium* (13.7–20.4%), *Pseudomonas* (2.1–12.7%), *Pseudarthrobacter* (2.6–6.6%), *Mycobacterium* (2.2–3.5%), *Sphingomonas* (1.6–3.7%), *Novosphingobium* (1.8–3.6%), *Massilia* (0.4–4.5%), *Bacillus* (0.2–5.1%), *Gemmatimonas* (1.5–2.7%), and *Flavobacterium* (0.3–4.7%).

Compared with the control, all *S. terrestris* treatments elevated the relative abundances of *Bradyrhizobium* and *Sphingomonas*; the LF treatment increased those of *Pseudomonas* and *Gemmatimonas*; the SF treatment increased the relative abundances of *Bacillus* and *Flavobacterium*, while decreasing those of *Novosphingobium* and *Gemmatimonas*. Fungi at the genus level mainly included 6 groups.

Identifiable genera and their relative abundances were: *Mortierella* (40.7–42.8%), *Cryptococcus* (26–29.9%), *Exophiala* (7.6–11.3%), *Bifidobacterium* (5.7–9.9%), and *Paraglomus* (5.8–6.4%).

Compared with the control, different treatments with *S. terrestris* both increased the relative abundance of *Mortierella*; the LF treatment increased the relative abundances of *Cryptococcus* and *Paraglomus*; the SF treatment increased the relative abundances of *Exophiala* and *Bifidobacterium* (Figure 9).

#### 3.3.4. α Diversity Analysis of Bacteria and Fungi

The OTU coverage rates of bacteria and fungal communities were over 99.6% and 100%, respectively. For bacteria, the Shannon, Simpson, Chao1, and ACE index in the LF and SF groups were significantly higher than those in the CK group. The Shannon index, Chao1 index, and ACE index in the SF group were slightly higher than those in the LF group, with no significant difference between the two groups. In the case of fungi, the Shannon and Simpson index in the LF group were significantly reduced compared to the CK group, whereas the Shannon index in the SF group showed a marginal increase relative to the CK group, with no significant variation in the Simpson index between the SF group and the CK group. Both the Chao1 index and ACE index in the LF and SF groups were significantly higher than those in the CK group, with the SF group exhibiting significantly higher values for these two indices than the LF group (Table 2).

In summary, the application of different fermentates of *S. terrestris* can alter the α diversity of the rhizosphere microbiota of understory *P. notoginseng*.

#### 3.3.5. β Diversity Analysis of Rhizosphere Soil Microorganisms

**Figure 10:**
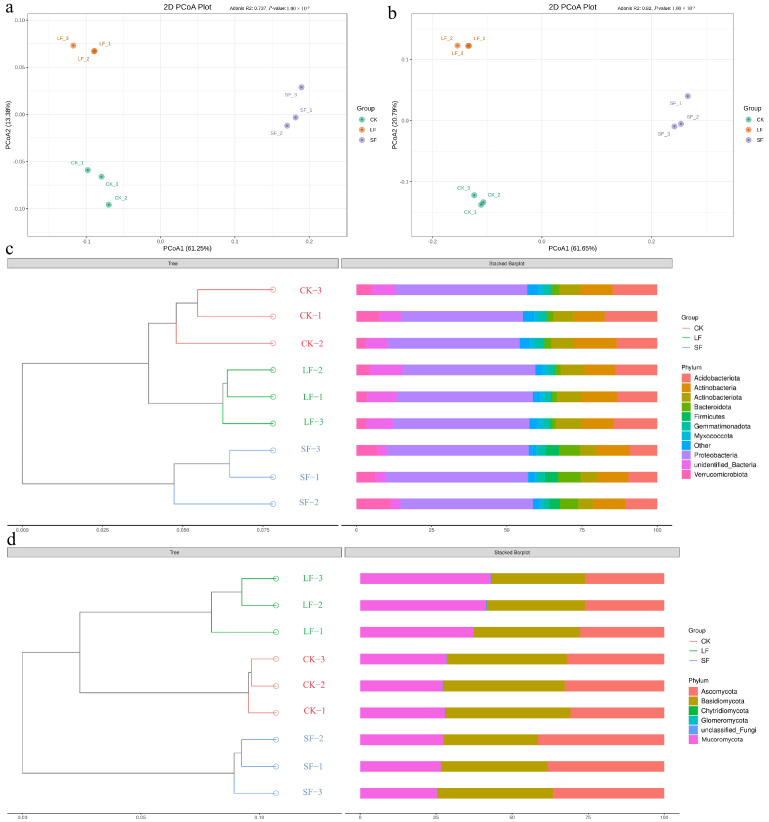
Impact of Different Treatments on *β*-Diversity of Rhizosphere Microorganisms. (**a**) Principal Coordinate Analysis of Bacteria, (**b**) Coordinate Analysis of Fungi, (**c**) UPGMA Clustering Tree Structure of Bacteria, (**d**) UPGMA Clustering Tree Structure of Fungi.

Weighted UniFrac-based PCoA (Figure 10) revealed that distinct *S. terrestris* treatments markedly reshaped the rhizosphere microbial community structure of understory *P. notoginseng*: For the soil bacterial community, PC1 and PC2 accounted for 61.25% and 13.38% of variance, respectively. LF and CK groups were clearly segregated from the SF group along PC1 with a large dissimilarity, and all treatments were separated along the second coordinate axis. For the soil fungal community, PC1 explained 61.65% of variance and PC2 20.79%. The treatment groups were widely dispersed across axes, indicating marked dissimilarity of community composition.

In summary, distinct *S. terrestris* treatments exerted a significant impact on the rhizosphere community, modifying the proportion of dominant microorganisms in understory *P. notoginseng* rhizosphere.

The Simpson index can serve as an important indicator reflecting community evenness (the closer the value is to 1, the higher the community evenness). The results show that: For the bacterial community: The Simpson index of the SF group (0.9933) was significantly higher than that of the CK group (0.9910, *p* < 0.05), indicating that SF treatment significantly improved the evenness of the bacterial community, which is consistent with the speculation that “SF promotes a more balanced microbial community structure”. For the fungal community: There was no significant difference between the Simpson index of the SF group (0.9560) and that of the CK group (0.9583). However, the Chao1 and ACE indices (richness indicators) of the SF group were significantly higher than those of the CK group (*p* < 0.05), suggesting that SF increased the species richness of fungi without significantly reducing community evenness. In contrast, the slightly higher evenness in the CK group might be attributed to the high abundance of a few dominant taxa (such as specific pathogenic fungi).

#### 3.3.6. Network Stability Analysis

**Figure 11:**
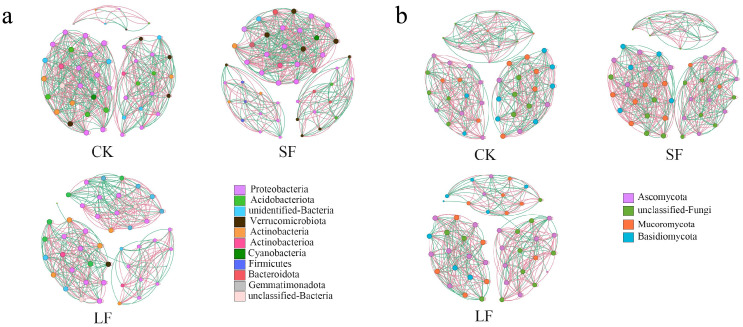
Microbial correlation analysis network diagram. (**a**) Correlation Analysis Network Diagram of Bacteria with the Top 50 Relative Abundances, (**b**) Correlation Analysis Network Diagram of Fungi with the Top 50 Relative Abundances.

Soil microbial interactions underpin biological activities such as “linguistic” communication and signal transduction, which are pivotal for sustaining soil microecological equilibrium. The top 50 ASVs (Amplicon Sequence Variants) by relative abundance, encompassing bacteria and fungi, were selected for network construction (Figure 11). Network analysis showed that distinct treatments with the endophytic fungus *S. terrestris* increased node count, edge number, graph density, and average clustering coefficient of the soil bacterial–fungal network, with a trend of SF > LF > CK. This indicates that distinct *S. terrestris* treatments augmented the network complexity of rhizosphere microorganisms in understory *P. notoginseng* (Table 3 and Table 4).

#### 3.3.7. Analysis of Environmental Factors Affecting Microbial Communities

**Figure 12:**
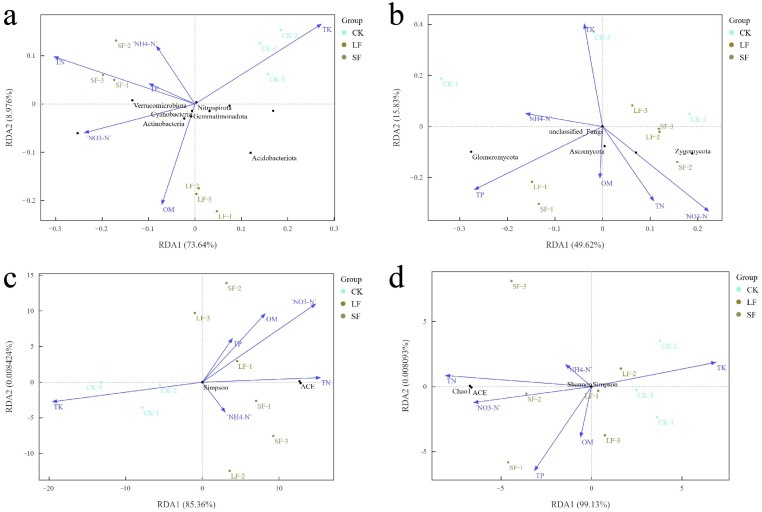
Effects of environmental factors on microbial composition and diversity. (**a**) Impact of Environmental Factors on Bacterial Composition, (**b**) Impact of Environmental Factors on Fungal Composition, (**c**) Impact of Environmental Factors on Bacterial Diversity, (**d**) Impact of Environmental Factors on Fungal Diversity.

Redundancy analysis (RDA) was conducted to dissect associations between soil physicochemical properties (total N, total P, total K, NH_4_^+^-N, NO_3_^−^-N, organic matter, pH) and *P. notoginseng* rhizosphere microorganisms, with results presented in Figure 12. Vector arrow length and direction in the RDA ordination plot indicate the correlation between microbial communities and soil chemical properties. For bacterial phyla, soil factors collectively explained 82.61% of variance: total N, total P, and NH_4_^+^-N were positively correlated with Verrucomicrobia; organic matter correlated positively with Actinobacteria and Gemmatimonadetes. Among these, total nitrogen and organic matter emerged as core drivers of bacterial phylum communities, with the broadest influence. For fungal phyla, the total explanatory power was 65.45%: total nitrogen showed a positive correlation with Glomeromycota; nitrate nitrogen was positively correlated with Mucoromycota; organic matter was positively correlated with Ascomycota. Total nitrogen and nitrate nitrogen were the core drivers shaping fungal phylum community structure, exerting the most extensive influence. For bacterial diversity (ACE, Chao1, Shannon, and Simpson indices), soil factors collectively explained 85.37% of variance; total nitrogen and ammonium nitrogen correlated positively with ACE and Simpson indices, with total N as the dominant factor exhibiting the broadest influence. For fungal diversity (involving ACE, Chao1, Shannon, and Simpson indices), the total explanatory rate was as high as 99.13%; total nitrogen and nitrate nitrogen were positively correlated with ACE and Chao1 indices, both exerting considerable influences. In summary, soil total nitrogen exerted the strongest overall influence on *P. notoginseng* rhizosphere microorganisms, encompassing bacterial phyla, fungal phyla, and their diversity.

## 4. Discussion

### 4.1. Effects of Growth-Promoting Fungi on Root Exudates

Rhizosphere soil metabolites, pivotal mediators of material exchange between plant roots and the external environment, directly mirror rhizosphere microecological equilibrium through their composition and dynamic fluctuations [33]. Root-released organic compounds (carbohydrates, organic acids, amino acids [34]) not only provide energy and nutrients for rhizosphere microbial survival but also modulate microbial community structure and function through signal transmission, thereby influencing soil nutrient transformation efficiency and plant growth [35,36]. Ecologically, they function as “regulators” of the rhizosphere microenvironment: by altering microbial activity and facilitating material cycling, they indirectly boost plant nutrient uptake capacity and stress tolerance [24]. In this study, the changes in chlorophyll content and other biomass-related traits of the plants may be associated with the growth-promoting functions of the fungus. Endophytic fungi with growth-promoting capabilities, such as phosphate-solubilizing and IAA-producing activities, can promote plant growth, thereby altering plant biomass [37,38].

Exogenous microbial introduction typically disturbs the inherent balance of rhizosphere metabolism. Prior studies have verified that microbial inoculation can markedly alter the types and contents of rhizosphere metabolites via their own metabolism or by inducing plant root secretion [39]. These changes are non-random, reflecting synergistic environmental adaptation by microorganisms and plants. For example, endophytic fungi enhance host resistance to adverse conditions by regulating metabolite synthesis [40,41]. In this study, distinct *S. terrestris* treatments differentially affected *P. notoginseng* rhizosphere metabolism, implying that SF may have stimulated the activity of rhizosphere microorganisms through more efficient metabolite release or signal transmission, thereby improving the overall metabolic level. The differential effects of SF and LF treatments of *S. terrestris* on rhizosphere metabolism may be attributed to the regulatory differences in microbial colonization, metabolite release, and microenvironmental interactions caused by formulation characteristics. The solid matrix of SF (e.g., rice husk) provides physical protection and attachment surfaces for fungi; its porous structure simulates the soil microenvironment, reduces interference, and improves colonization stability. In contrast, the free spores or hyphae in LF are susceptible to adsorption by soil particles and dilution by water, resulting in lower colonization efficiency. Meanwhile, SF achieves the sustained release of metabolites (such as enzymes and organic acids) through the slow degradation of the matrix, forming a stable rhizosphere metabolic gradient, whereas the metabolites in LF sharply decrease in concentration due to rapid diffusion, making it difficult to exert continuous effects. This difference enables SF to more effectively stimulate microbial activity: the complex organic matter in the solid matrix induces fungi to preferentially synthesize extracellular enzymes, enhancing the coupling with soil nutrient cycles. Previous studies support this pattern. Recent research findings have shown that solid fermentation (SF) enhances microbial colonization ability, enzyme activity, and long-term biological functions through structural stability, sustained release of metabolites, and compatibility with the microenvironment of rhizosphere ecology. In contrast, although liquid fermentation (LF) can rapidly produce metabolites, its efficacy is limited due to environmental sensitivity and transient interactions [42,43,44].

Metabolic pathway shifts are pivotal for deciphering rhizosphere metabolic regulation. As ubiquitous “material transporters”, ABC transporters mediate secondary metabolite transport, potentially accelerating the transfer and utilization of beneficial rhizosphere substances [45]. Meanwhile, purine metabolism activation supports the rhizosphere microecology in two aspects: energy supply and nitrogen source utilization. Purine substances ensure energy supply by participating in ATP synthesis, with their degradation products serving as nitrogen sources by plants [46,47]. *Setophoma terrestris*-mediated regulation of these pathways essentially entails optimizing rhizosphere material and energy metabolism to construct a more favorable microenvironment for *P. notoginseng* growth. This also provides a metabolic-level theoretical basis for using microorganisms to regulate plant growth. The identification of metabolites in this study focuses on the overall trends of major categories. The structural confirmation of individual compounds requires further experimental support; however, the functions of these categories (such as purines and saponins) are consistent with those reported in existing studies, which does not affect the overall conclusion. Furthermore, structurally related compounds may exhibit similar biological activities. For instance, homologs of notoginsenoside R1 may be synchronously reduced to alleviate autotoxicity, and analogs of purine metabolites may synergistically participate in energy and nitrogen metabolism. This hypothesis requires verification through subsequent in vitro activity assays.

### 4.2. Effects of Growth-Promoting Fungi on Soil Chemical Properties

Soil chemical properties are core indicators of soil fertility, with microorganisms driving their improvement [48]. In natural ecosystems, microorganisms decompose organic matter and transform nutrient forms, converting unavailable substances into plant-absorbable forms. Meanwhile, they contribute to humus formation, boosting soil water- and nutrient-retention capacities [49,50]. This role is not isolated but interfaces with soil physical structure and biological communities to collectively maintain soil health.

Soil chemical property improvement by growth-promoting fungi stems fundamentally from the interaction between their metabolic activities and the soil environment [51]. They secrete enzymes to decompose organic substances, releasing elements (e.g., nitrogen, carbon) to elevate soil nutrient levels. Additionally, their metabolites may regulate soil colloid structure, affect physicochemical parameters such as pH, and indirectly improve soil aeration and nutrient retention [52]. From an agricultural application standpoint, such microorganism-mediated soil improvement is sustainable. Relative to chemical fertilizers, microorganisms modulate soil properties via natural metabolic processes, avoiding nutrient loss and environmental pollution while sustaining soil fertility balance through long-term effects [53].

*Setophoma terrestris*-mediated improvement of *P. notoginseng* rhizosphere soil aligns with the functional pattern of most growth-promoting fungi. Its processes of increasing soil nitrogen content and organic matter, as well as regulating pH, reflect the dynamic coupling between microorganisms and the soil nutrient pool: organic nitrogen is decomposed into available forms, while soil organic matter is supplemented via its residues and metabolites, forming a closed “microorganism-soil-plant” nutrient cycle. This improvement entails not the enhancement of a single indicator but the synergistic optimization of overall soil fertility, furnishing a stable nutrient supply for plant growth.

### 4.3. Effects of Growth-Promoting Fungi on Rhizosphere Soil Microbial Community Structure

Soil microbial communities are core components of the rhizosphere microecology, and their structural diversity and functional stability directly determine the service capacity of soil ecosystems [54]. Exogenous microbial introduction functions as a “regulator” in the community, reshaping the original community structure through resource competition, signal crosstalk, or microenvironment modification [55]. This reshaping entails not simple species replacement but community function optimization via enriching beneficial microorganisms and inhibiting harmful populations. For example, increased abundance of nutrient-transforming bacterial genera may improve soil nutrient use efficiency, while elevated disease-resistant microbial abundance can enhance plant resistance to diseases [56].

Growth-promoting fungal regulation of microbial communities adheres to clear ecological logic: they may inhibit pathogens via antimicrobial secretion or expand ecological niches by forming symbiotic relationships with beneficial microorganisms, ultimately establishing a more stable community structure [57]. In terms of diversity, increased community richness and evenness denote stronger environmental adaptability. Diverse microbial groups can perform different ecological functions, circumventing functional imbalance arising from environmental fluctuations affecting a single species [58]. Meanwhile, shifts in microbial communities and improvements in soil physicochemical properties often form a positive feedback loop: optimized community structure promotes nutrient transformation, and elevated nutrient content provides more abundant resources for microorganisms, further reinforcing community stability. In this study, Glomeromycota, a phylum encompassing typical arbuscular mycorrhizal fungi (AMF), was detected at the fungal phylum level. At the genus level, AMF-related taxa such as *Paraglomus* were also observed. Among the treatment groups, the liquid fermentate (LF) significantly increased the relative abundance of *Paraglomus*, suggesting that *S. terrestris* may indirectly affect nutrient uptake in *P. notoginseng* by regulating AMF communities. It should be noted that this study did not determine the colonization rate of AMF in roots (e.g., hyphal infection rate). *Subsequent studies* could supplement this data through microscopic observation to more accurately clarify the role of AMF in the growth-promoting process of *S. terrestris*.

*Setophoma terrestris*-mediated regulation of *P. notoginseng* rhizosphere microorganisms reflects the synergistic adaptation between endophytic fungi and the host rhizosphere. Enriched phyla (e.g., Proteobacteria, Basidiomycota) are pivotal for nutrient uptake and organic matter decomposition, whereas reduced Acidobacteria abundance may correlate with elevated soil pH, indicative of microbial community adaptation to environmental shifts. This microbial-mediated community optimization ultimately provides ecological support for *P. notoginseng* growth by improving soil nutrient cycling efficiency and microenvironment stability, while offering practical insights into plant–microbe interaction mechanisms.

Since this experiment was conducted in a field, the results of this study are highly reproducible in the main production areas of Panax notoginseng (with similar soil and climate conditions). In the future, multi-location verification can be carried out to test the stability under different environments.

## 5. Conclusions

Exogenous application of *S. terrestris* markedly enhanced the growth of *P. notoginseng*, as evidenced by increased chlorophyll and total saponin contents, and improved plant height and fresh/dry weights.

It profoundly affected *P. notoginseng* rhizosphere soil metabolism: SF treatment exhibited a higher metabolic level relative to LF treatment.

In total, 1635 differential metabolites (892 upregulated) were identified in SF vs. CK, whereas 553 (171 upregulated) were detected in LF vs. CK. This fungus reduced pathogenic toxic metabolites (e.g., enniatin) and harmful components (e.g., notoginsenoside R), while increasing the abundance of key purine pathway products (e.g., adenine).

In terms of soil improvement, *S. terrestris* augmented soil fertility: both treatments increased total nitrogen content and soil pH, with the SF treatment significantly elevating ammonium nitrogen and the LF treatment markedly increasing organic matter.

Notable soil microbial community regulatory effects were observed: it increased the abundance of dominant groups (e.g., bacterial Proteobacteria, fungal Mucoromycota) and elevated the proportions of nitrogen-fixing bacteria (e.g., Bradyrhizobium) and Mortierella. Additionally, it significantly altered microbial richness and enhanced bacterial–fungal network complexity (evidenced by increased node and edge counts).

Soil total nitrogen, ammonium nitrogen, and organic matter were recognized as key drivers shaping microbial communities, thereby substantiating the mechanism underpinning *S. terrestris*-mediated growth promotion in *P. notoginseng*.

## Figures and Tables

**Table 1:** Effects of different treatments on soil physicochemical properties.

Treatments	Total Nitrogen (g/kg)	Total Phosphorus (g/kg)	Total Potassium (g/kg)	Ammonium Nitrogen (mg/kg)	Nitrate Nitrogen (mg/kg)	Organic Matter (g/kg)	pH
LF	1.118 ± 0.05 b	1.388 ± 0.01 a	12.124 ± 0.03 a	8.048 ± 0.34 a	57.174 ± 7.8 ab	16.09 ± 0.44 a	6.647 ± 0.12 a
SF	1.32 ± 0.02 a	1.395 ± 0.01 a	12.104 ± 0.03 a	9.102 ± 0.98 a	63.9 ± 3.82 a	13.702 ± 1.35 ab	6.413 ± 0.18 a
CK	1.09 ± 0.02 b	1.388 ± 0.01 a	12.298 ± 0.03 a	8.69 ± 2.05 a	45.316 ± 9.59 b	13.691 ± 0.9 b	6.01 ± 0.19 b

Note: Different letters after data indicate significant differences (*p* < 0.05). SF refers to the solid fermentation product treatment group, LF refers to the fermentation broth treatment group, and CK refers to the clear water treatment control group. The same applies to the tables below.

**Table 2:** Effects of different treatments on the diversity α rhizosphere microbial.

Microbe	Treatment	Richness Index		Diversity Index		Degree of Coverage
		Chao1	ACE	Shannon	Simpson	
bacterial	LF	1684.1727 ± 38.7319 a	1689.1887 ± 36.5441 a	6.2883 ± 0.0015 a	0.993 ± 0 a	0.9967 ± 0.0006 a
	SF	1736.6377 ± 40.9896 a	1741.3617 ± 37.73 a	6.3973 ± 0.087 a	0.9933 ± 0.0012 a	0.9967 ± 0.0006 a
	CK	1540.1837 ± 50.6641 b	1546.4637 ± 49.9319 b	6.1697 ± 0.0424 b	0.991 ± 0 b	0.9967 ± 0.0006 a
fungi	LF	340.985 ± 4.2508 b	341.9047 ± 4.3635 b	3.7527 ± 0.0533 b	0.9483 ± 0.0012 b	1
	SF	375.525 ± 3.5996 a	375.8447 ± 3.5807 a	3.9627 ± 0.0482 a	0.956 ± 0.003 a	1
	CK	324.9853 ± 4.7512 c	325.9927 ± 4.7811 c	3.8853 ± 0.052 a	0.9583 ± 0.0025 a	1

Note: Different letters after data indicate significant differences (*p* < 0.05).

**Table 3:** Topological properties of bacterial community co-occurrence network with different treatments.

Topological Properties	CK	LF	SF	Topological Properties	CK	LF	SF
Node	48	48	49	Network diameter	1	1	1
Edge	388	492	469	Component	6	5	3
Negative correlations	40.5%	45.5%	44%	Modularity	3.047	7.251	2.853
Average degree	15.6	20.5	19.14	Average clustering coefficient	0.973	0.981	0.979
Graph density	0.318	0.436	0.399	Average path	1	1	1

**Table 4:** Topological properties of fungi community co-occurrence network with different treatments.

Topological Properties	CK	LF	SF	Topological Properties	CK	LF	SF
Node	48	49	50	Network diameter	1	1	1
Edge	388	429	468	Component	6	4	3
Negative correlations	40.5%	43%	40.6%	Modularity	3.155	6.115	2.026
Average degree	15.6	17.2	18.7	Average clustering coefficient	0.973	0.974	0.977
Graph density	0.318	0.351	0.382	Average path	1	1	1

## Data Availability

The data from this trial can be found in the document. Reagents, microbial materials, and datasets used, created, and analyzed in the study are available upon request to the corresponding author. The genomic sequencing data mentioned in this study are stored in the Sequence Read Archive (SRA) database of the National Center for Biotechnology Information (NCBI) and can be accessed via the NCBI database. The source data are presented in the form of additional source datasets.
